# Predicting ordinal clinical outcomes in at-risk mental states: a multimodal approach

**DOI:** 10.3389/fpsyt.2026.1808209

**Published:** 2026-05-28

**Authors:** Kazuya Nagasawa, Yuko Higuchi, Naohito Kaneko, Kensei Miyazu, Shunsuke Shimataki, Shimako Nishiyama, Yukiko Akasaki, Marino Izumi, Noa Tsujii, Tsutomu Takahashi

**Affiliations:** 1 Department of Neuropsychiatry, University of Toyama Graduate School of Medicine and Pharmaceutical Sciences, Toyama, Japan; 2 Research Center for Idling Brain Science, University of Toyama, Toyama, Japan; 3 Center for Health Care and Human Sciences, University of Toyama, Toyama, Japan; 4 Department of Child Mental Health and Development, Toyama University Hospital, Toyama, Japan

**Keywords:** at-risk mental state, cognition, event-related potential, mismatch negativity, prediction, psychosis, schizophrenia

## Abstract

**Introduction:**

Clinical outcomes in individuals with at-risk mental states (ARMS) are heterogeneous and extend beyond the simple dichotomy between transition and non-transition to psychosis. While previous studies have primarily focused on predicting the transition to psychosis, few have systematically examined multiple outcome stages, including remission and persistent subthreshold symptoms, using integrated neurobiological markers. This study aimed to identify the predictors of multilevel clinical outcomes in ARMS using a multimodal framework that incorporates clinical, functional, and electrophysiological measures.

**Methods:**

Eighty-seven subjects with ARMS were included and followed up, and the clinical outcomes were classified into four ordered categories based on a framework derived from the North American Prodrome Longitudinal Study 2 (NAPLS-2): remission, symptomatic, prodromal progression, and psychotic. Ordinal logistic regression analyses were conducted to identify predictors associated with ordered clinical outcomes using baseline measures as candidate predictors. Fifteen explanatory variables were used, including clinical symptoms, cognitive functioning, and electrophysiological measures [amplitudes and latencies of P300, duration mismatch negativity (dMMN), and frequency MMN (fMMN)].

**Results:**

Reduced baseline dMMN amplitude, greater severity of attenuated positive symptoms indexed by unusual thought content, and poor cognitive functioning associated with daily living, assessed using the Schizophrenia Cognition Rating Scale, were independently associated with worse ordered clinical outcomes.

**Discussion:**

These findings suggest that future clinical trajectories of ARMS can be predicted by multimodal factors spanning neurophysiological, clinical, and functional domains. Early stratification of individuals at the ARMS stage may contribute to the development of personalized and stage-appropriate intervention strategies tailored to subsequent clinical outcomes.

## 1 Introduction

Schizophrenia—one of the most common psychiatric disorders that develop in adolescence and early adulthood—often follows a chronic course accompanied by significant functional decline. Delays in treatment initiation have been shown to adversely affect clinical outcomes, highlighting the importance of early diagnosis and appropriate interventions (1). In this context, the at-risk mental state (ARMS) for developing psychosis, including schizophrenia, has been operationally defined based on distinctive clinical features and is considered an important target for early detection and preventive interventions (2).

Only a proportion of individuals diagnosed with clinical high risk for psychosis (CHR-P), a concept largely overlapping with ARMS, subsequently develop a psychotic disorder. In line with this observation, a large-scale meta-analysis of 130 studies including 9,222 individuals reported cumulative transition rates of approximately 15% at 1 year, 25% at 3 years, and 35% at 10 years after initial CHR-P identification (3). Importantly, non-transition to psychosis does not necessarily indicate a favorable clinical outcome. A meta-analysis focusing on non-transitioned individuals showed that only 48.7% no longer met the CHR-P criteria at a mean follow-up of approximately 30.7 months, whereas subthreshold symptoms persisted in a substantial proportion of these individuals (4). Consistent with these findings, prospective cohort studies have demonstrated that individuals who do not transition may exhibit diverse clinical trajectories, including persistent symptoms and the emergence of other psychiatric disorders, such as mood, anxiety, and substance use disorders, rather than exhibiting sustained clinical recovery (5). These findings suggest that the clinical outcomes in ARMS/CHR-P populations are heterogeneous and cannot be fully captured by a simple dichotomy between transition and non-transition.

Despite extensive research on outcome prediction in individuals with ARMS/CHR-P, predictors supported by consistent and robust evidence remain limited (6). Most previous studies have disproportionately focused on transition to psychotic disorders as the primary outcome, with predictive factors largely restricted to clinical and functional measures, including attenuated psychotic symptoms, negative symptoms, functioning deficits, and neurocognitive deficits (6). By contrast, remission—arguably one of the most clinically meaningful outcomes among non-transitioned individuals—has been relatively understudied. Even in studies that examined remission, analyses primarily relied on baseline clinical and functional measures and only a limited number of independent predictors were identified (7, 8), underscoring the need for more comprehensive and integrative predictive models.

Among candidate predictive biomarkers, event-related potentials (ERPs), particularly mismatch negativity (MMN), have attracted increasing attention. ERPs are neurophysiological measures derived from electroencephalography that allow assessment of neural information processing with high temporal resolution (9). MMN is an auditory ERP elicited by deviations from regular auditory patterns, such as duration or frequency, and is considered to reflect pre-attentive sensory processing without requiring active task engagement (10). Accumulating evidence indicates that reduced MMN amplitude is a robust finding in schizophrenia and has been associated with functional recovery and prognosis (11). Importantly, similar MMN abnormalities have also been observed in individuals with ARMS/CHR-P, suggesting that MMN-related dysfunction is already present prior to the onset of overt psychotic disorders (12). Comprehensive reviews of CHR-P research have identified electrophysiological markers, including MMN, as predictors of later transition to psychosis (6). Notably, a few studies have extended beyond transition to psychosis and examined non-transition outcomes in ARMS/CHR-P populations; baseline MMN measures have been reported to predict subsequent symptomatic and functional improvement (13) as well as neurocognitive functioning (14) in non-transitioned individuals. However, most previous findings were derived from exploratory studies that relied on dichotomous outcome definitions. Consequently, studies that simultaneously address multiple ordered outcome stages while integrating electrophysiological measures with established clinical indicators remain limited.

One important methodological issue in studies investigating neurophysiological biomarkers in ARMS/CHR-P populations is whether participants are receiving antipsychotic medication at baseline. While antipsychotic medication is not generally recommended as a first-line preventive intervention for individuals at ARMS/CHR-P, clinical guidelines allow cautious use of antipsychotics for crisis intervention when clinically necessary, such as in cases of acute worsening of psychotic symptoms, marked distress with strong help-seeking, or significant risk requiring rapid stabilization (15, 16). Although the MMN measures employed in the present study are considered to be relatively stable under antipsychotic treatment (17), the potential influence of antipsychotic medication cannot be completely excluded in real-world clinical cohorts. Therefore, methodological approaches such as subgroup analyses restricted to drug-free samples and statistical adjustments for antipsychotic exposure (e.g., medication status or dosage) may be necessary.

This study aimed to identify predictors of long-term clinical outcomes in individuals with ARMS using a multimodal approach that integrates electrophysiological measures with established clinical and functional indicators. Rather than a simple dichotomy between transition and non-transition to psychosis, clinical outcomes were classified into several ordered categories reflecting remission, symptom persistence, progression of prodromal symptoms, and transition to psychosis. Outcome stratification was based on the framework developed in the North American Prodrome Longitudinal Study 2 (NAPLS-2), as described below. Within this ordinal outcome framework, baseline ERP indices, including MMN, were examined alongside clinical and functional measures as candidate outcome predictors. We hypothesized that ERP indices, particularly MMN measurements, may serve as electrophysiological biomarkers that contribute to the prediction of multiple ordered clinical outcomes in individuals with ARMS. In this context, participants receiving antipsychotic medication at baseline were retained in the analysis to reflect real-world clinical practice and to preserve statistical power given the limited sample size of the cohort, and additional statistical adjustments were performed.

## 2 Materials and methods

### 2.1 Participants

A total of 87 subjects with ARMS (45 males and 42 females; mean ± SD: 18.3 ± 4.3 years), recruited from the Consultation Support Service in Toyama (CAST), which is a local clinical setting specialized in early interventions (18), participated in this study. Individuals with ARMS were identified by experienced psychiatrists or clinical psychologists using the Comprehensive Assessment of At-Risk Mental State (CAARMS) (19). ARMS subgroups included attenuated psychotic symptoms, genetic risk and deterioration syndrome (GRD), and brief and limited intermittent psychotic symptoms, as defined by the CAARMS. Eligible participants were confirmed to have good hearing and physical health based on physical examinations and standard laboratory tests. Individuals were excluded if they had a history of substance abuse or dependence, seizures, or head injury. The mean the Japanese Adult Reading Test (JART) (20) score was 97.1, and none of the subjects had a clinical diagnosis of intellectual disability. Of the 87 subjects with ARMS, 15 received antipsychotic medication (mean ± SD: 0.2 ± 0.7 mg/day, risperidone equivalent), while the remaining 72 were either antipsychotic-naïve or had been free from antipsychotic treatment for at least 2 weeks (21).

After the baseline assessments, participants were prospectively followed up for a mean duration of 964.1 ± 964.6 (range: 172–3830) days, during which clinical outcomes were evaluated based on CAARMS assessments.

The Committee on Medical Ethics of the University of Toyama approved this study protocol (approval no. I2013006; February 5, 2014, and R2023213; January 4, 2024). This study was conducted in accordance with the principles of the Declaration of Helsinki. Written informed consent was obtained from all the participants. For minor participants, written informed consent was obtained from their parents or legal guardians.

### 2.2 Clinical assessment

Experienced psychiatrists or clinical psychologists evaluated the clinical symptoms of individuals with ARMS using the CAARMS and the Positive and Negative Syndrome Scale (PANSS) (22) at baseline. The Brief Assessment of Cognition in Schizophrenia (BACS) Japanese version (23, 24), Schizophrenia Cognition Rating Scale Japanese version (SCoRS-J) (25, 26) and modified Global Assessment of Functioning (mGAF) (27) were used to evaluate each participant’s cognitive and social functioning.

Follow-up CAARMS and PANSS assessments were systematically conducted at prespecified follow-up timepoints defined in advance at our institution.

### 2.3 Outcome classification

The clinical outcomes for individuals with ARMS were classified into four outcome groups based on the definitions proposed in the NAPLS-2 (28). In the original NAPLS-2 study, ARMS status was assessed using the Scale of Prodromal Symptoms (SOPS). The SOPS consists of five positive symptom domains: Unusual Thought Content/Delusional Ideas (P1), Suspiciousness/Persecutory Ideas (P2), Grandiose Ideas (P3), Perceptual Abnormalities/Hallucinations (P4), and Disorganized Communication (P5) (29). At each follow-up assessment, clinical outcomes were determined as follows: (i) remission was defined as remission from all prodromal syndromes, indicated by scores of 2 or less on all five positive symptoms of the SOPS scale; for those who have only GRD, “remission” required recovery of the Global Assessment of Functioning (GAF) score to at least 90% of previous best level; (ii) symptomatic was defined as not currently meeting criteria for a prodromal risk syndrome but having ratings of 3 to 5 on any one of the five positive symptoms of the SOPS, or showing no improvement in the GAF from baseline; (iii) prodromal progression was defined as currently meeting criteria for ARMS; and (iv) psychotic was defined as currently meeting criteria for a psychotic disorder or evidencing scores of 6 on one or more positive symptoms of the SOPS. In the present study, outcome classification followed the conceptual framework of the NAPLS-2, with clinical assessments conducted using the CAARMS, which is conceptually comparable to the SOPS. While the CAARMS and SOPS differ in their specific operational criteria, they assess overlapping prodromal symptom constructs with no major conceptual discrepancies. One notable difference between these two instruments is that grandiosity is operationalized as an independent positive symptom item in SOPS P3, whereas in CAARMS 2006 version applied in the present study, it is assessed within the broader non-bizarre ideas domain. Therefore, the PANSS grandiosity item was used as a proxy for SOPS P3, given the conceptual overlap in assessing grandiose ideation within the positive symptom domain. Detailed information on the assessment and scoring is provided in **Supplementary Table 1**. In this study, (i) remission, (ii) symptomatic, (iii) prodromal progression, (iv) psychotic, were referred to as “Outcome 1,” “Outcome 2,” “Outcome 3,” and “Outcome 4,” respectively.

### 2.4 ERP recording

ERPs were recorded using an auditory oddball paradigm based on an established method used at our institute (30–34). Briefly, electroencephalogram (EEG) recordings were obtained using either a Nihon Kohden EEG device (EEG-1250 version 07-02, Nihon Kohden Corp., Tokyo, Japan) or Polymate AP1532 (TEAC Corp., Tokyo, Japan) and either a 32-channel Electrocap (Electrocap Inc., Eaton, OH, USA) or 32-channel MCS cap (Medical Computer Systems Ltd., Zelenograd, Moscow, Russia) in a wave-shielded and sound-attenuated room. Auditory stimuli were delivered binaurally using headphones. P300 recordings were conducted with the participants lying awake on a bed, keeping their eyes open while watching a red circle on a display monitor. The participants were observed carefully, and if they were in poor condition (asleep, too many eye blinks or eye movements, frequent body movements, or unwillingness to participate in the examination), we repeated the instructions or stopped the recording. By contrast, during the MMN recording, the participants were seated while watching a silent cartoon to help them stay alert without auditory interference. The auditory oddball paradigms were employed using duration- or frequency-deviant stimuli. For P300, 250 stimuli comprising 80% standard tones (1,000 Hz, 50 ms) and 20% deviant tones (2,000 Hz, 50 ms) were used. For dMMN, 1500 stimuli comprising 90% standard tones (1,000 Hz, 50 ms) and 10% deviant tones (1,000 Hz, 100 ms) were used. For fMMN, 1,500 stimuli comprising 90% standard tones (1,000 Hz, 50 ms) and 10% deviant tones (1,500 Hz, 50 ms) were used. For P300, auditory stimuli were delivered binaurally through headphones with variable inter-stimulus intervals ranging from 1.5–2.5 s, and for MMN measurements, the inter-stimulus interval (ISI) was fixed at 500 ms, resulting in a stimulus-onset asynchrony (SOA) of 550 ms for standard tones (50 ms) and 600 ms for dMMN deviant tones (100 ms). Auditory parameters were delivered at a 60-dB sound pressure level and a 10 ms rise/fall time. Data were collected at a sampling rate of 500 Hz. The bandwidth was set at 0.53–120 Hz using a 60 Hz notch filter. The reference electrode was located at Aav, and the ground electrode, at Z. Electrode impedance was less than 10 kΩ. The auditory stimuli were presented in three consecutive blocks: P300 (first), dMMN (second), and fMMN (third). There were approximately 1 min break times between the three blocks. Epochs were averaged using EPLYZER II (Kissei Comtec Co., Ltd., Nagano, Japan): 700 ms (P300), 600 ms (dMMN), or 500 ms (fMMN) epochs, each including a 100 ms pre-stimulus baseline. Epochs containing voltage excursions > ± 100 µV caused by blinks, eye movement, or body movement were manually discarded. Artifact-free epochs were averaged separately for target and non-target waveforms. For MMNs, the target waveforms were subtracted from the non-target waveforms to yield the MMN. Each epoch was baseline-corrected by subtracting the mean voltage in the −100 to 0 ms window. The amplitude and latency of the ERPs were used as parameters. The P300 amplitude and latency were defined using the positive peak occurring 250–400 ms after the stimulus onset. By contrast, dMMN was quantified using the negative peak, identified within 130–250 ms for dMMN and 60–180 ms for fMMN following stimulus onset. For statistical analyses, only the recordings at Pz for P300 and Fz for MMNs, which generally have the greatest amplitude compared with those at other electrodes, were used as representative ERPs for each individual, according to previous literature (35–38). Detailed data regarding the measurement conditions are provided in **Supplementary Table 2**.

### 2.5 Statistical analysis

The demographic and clinical variables were compared using SPSS Statistics version 25 (IBM Corp., Armonk, NY, USA). Means and standard deviations were calculated for continuous variables and frequencies and percentages for categorical variables. For MMN amplitude, the waveforms showed negative polarity in all participants; therefore, absolute values were used for statistical analysis. Group differences among the four outcome groups (Outcomes 1-4) were examined using one-way analysis of variance (ANOVA), followed by Bonferroni-corrected *post-hoc* comparisons. For categorical variables, χ² tests were used to assess group differences.

Regression analyses were conducted using Python (version 3.13.5; Python Software Foundation) with the statsmodels library (version 0.14.4; for statistical modeling) and the scikit-learn library (version 1.6.1; for machine learning algorithms). Although the proportion of missing data was relatively low (on average a few percent across variables), missing values were addressed using multiple imputation by chained equations (MICE), with 20 imputed datasets generated following established methodological recommendations (39–42). All candidate baseline predictors were initially considered for inclusion in regression models. To minimize multicollinearity, however, some predictors presumed to be highly correlated were removed *a priori* based on clinical and psychometric considerations. The final set of candidate predictors included sex, age, Unusual Thought Content, Non-Bizarre Ideas, Perceptual Abnormalities, Disorganized Speech, JART, BACS, P300 latency, P300 amplitude, dMMN latency, dMMN amplitude, fMMN latency, fMMN amplitude, and SCoRS. Grandiosity was excluded from the regression models because very few participants exhibited grandiosity at baseline, resulting in limited variability (**Supplementary Table 3**). Antipsychotic medication dose was also excluded, as only a small proportion of participants were receiving antipsychotic treatment and no significant group differences were observed.

In the primary analysis, the associations between baseline characteristics and long-term outcomes were evaluated using ordinal logistic regression under a proportional odds framework (43). The proportional-odds assumption was assessed by examining the consistency of regression coefficients across cumulative thresholds within each multiply imputed dataset (44, 45). The models were fitted using the OrderedModel class in statsmodels with a logit link function. Variable selection was performed separately for each of the 20 imputed datasets using a bidirectional stepwise procedure based on Akaike’s Information Criterion (AIC) (46). Following recommended MI-based selection strategies (47), predictors selected in at least 10 of the 20 imputations (majority rule) were retained in the final predictor set to improve selection stability after multiple imputation. These predictors were then fitted to all imputed datasets, and regression coefficients were pooled using Rubin’s rules with small-sample t approximations (48). Odds ratios (ORs), 95% confidence intervals (CIs), and two-tailed p values were calculated.

The model fit was evaluated using McFadden’s pseudo-R² and the information criteria (AIC and BIC) averaged across imputations. Model performance was additionally evaluated using the ordinal C-index, the multi-class Brier score, and calibration plots. Internal validation of model performance was performed using bootstrap resampling (200 resamples) to estimate optimism in model performance (44, 45). Statistical significance was defined as p < 0.05 (two-tailed).

## 3 Results

### 3.1 Characteristics of study population

The demographic and clinical characteristics of the individuals with ARMS are summarized in **Table 1**. Based on their final clinical outcomes, participants were classified as follows: 25 (28.7%) into the Outcome 1 group, 18 (20.7%) into the Outcome 2 group, 21 (24.1%) into the Outcome 3 group, and 23 (26.4%) into the Outcome 4 group. Among individuals classified as Outcome 4, diagnoses included schizophrenia (n = 19), delusional disorder (n = 1), brief psychotic disorder (n = 1), other specified schizophrenia spectrum and other psychotic disorder (n = 2), and major depressive disorder (n = 1). For those who had not transitioned to psychosis (Outcomes 1-3), outcomes were determined based on the most recent symptom ratings. There were no significant differences among these three groups in the number of days from baseline to outcome-determining symptom assessment. No significant group differences were observed in age, sex distribution, medication status, antipsychotic dosage (risperidone equivalent), or estimated pre-morbid IQ (JART). By contrast, baseline PANSS positive symptom scores as well as Suspiciousness and Disorganized Speech scores, as measured by CAARMS, were significantly lower in the Outcome 1 group than in the Outcome 2 group. Compared to the other three outcome groups, the Outcome 1 group showed significantly lower Unusual Thought Content scores. Regarding cognitive and social functioning measures, the Outcome 1 group showed significantly lower SCoRS scores than the other three outcome groups, whereas no significant group differences were observed in BACS scores. Although a significant overall group effect was detected for the mGAF scores, *post-hoc* analyses did not reveal significant differences between any of the groups.

**Table 1:** Demographic and clinical data.

Demographic/Clinical assessments	All n=87	Outcome 1 n=25	Outcome 2 n=18	Outcome 3 n=21	Outcome 4 n=23	Statistics^a^
age (year)	18.3(4.3)	17.9(4.7)	18.3(3.8)	18.4(4.1)	18.7(4.5)	F(3,83)=0.132, p=0.941
male/female	45/42	14/11	11/7	11/10	9/14	χ^2^ = 2.283, p=0.516
drug free/medication	72/15	21/4	15/3	18/3	18/5	χ^2^ = 0.486, p=0.922
antipsychotic dose, risperidone equiv. (mg/day)	0.2(0.7)	0.3(1.0)	0.3(0.9)	0.2(0.5)	0.2(0.3)	F(3,83)=0.230, p=0.875
JART	97.1(10.1)	97.9(9.7)	98.4(9.8)	97.6(11.0)	94.5(10.0)	F(3,82)=0.645, p=0.588
follow-up period (day)	964.1(964.6)	819(850.0)	1353 (1108.6)	803.5(911.0)	–	F(2,61)=2.108, p=0.130
PANSS : positive	11.8(3.4)	10.4(2.6)	13.8(3.9)	11.2(3.3)	12.4(3.0)	F(3,82)=4.378, p=0.006**, Outcome 1<2
: negative	16.6(6.2)	16.4(7.4)	17.8(5.7)	14.4(5.9)	18.0(5.2)	F(3,82)=1.494, p=0.222
: general psychopathology	30.5(8.1)	27.7(6.9)	34.0(9.1)	28.7(7.5)	32.4(8.1)	F(3,82)=3.033, p=0.034*
: total	58.9(15.3)	54.5(13.5)	65.6(16.4)	54.3(15.2)	62.8(14.3)	F(3,82)=3.164, p=0.029*
CAARMS : Unusual Thought Content	3.3(1.6)	2.2(1.7)	3.8(1.4)	3.7(1.5)	3.9(1.2)	F(3,82)=7.138,p<0.001**, Outcome 1<2,3,4
: Suspiciousness	3.4(1.3)	2.8(1.3)	3.9(1.4)	3.6(1.4)	3.6(0.9)	F(3,69)=2.975, p=0.038*, Outcome 1<2
: Perceptual Abnormalities	2.9(1.5)	2.1(1.6)	3.2(1.8)	3.1(1.4)	3.2(1.3)	F(3,81)=2.868, p=0.042*
: Disorganized Speech	2.7(1.3)	2.1(1.3)	3.2(1.2)	2.4(1.1)	3.0(1.4)	F(3,81)=3.324, p=0.024*, Outcome 1<2
SCoRS^b^	5.4(2.2)	4.1(1.6)	5.8(2.3)	6.0(2.0)	5.9(2.2)	F(3,80)=4.634, p=0.005**, Outcome 1<2,3,4
mGAF^c^	41.5(8.8)	46.5(7.8)	38.9(8.8)	39.1(8.6)	41.7(8.5)	F(3,68)=2.993, p=0.037*
BACS^d^	-0.7(0.9)	-0.4(0.8)	-0.8(1.2)	-0.6(0.9)	-1.0(0.9)	F(3,83)=1.634, p=0.188

Values are shown as means (standard deviations).

Outcome 1 corresponds to “remission”, Outcome 2 to “symptomatic status”, Outcome 3 to “prodromal progression”, and Outcome 4 to “transition to psychosis”, according to the NAPLS-2 criteria [28]. BACS, Brief Assessment of Cognition in Schizophrenia; CAARMS, Comprehensive Assessment of At-Risk Mental States; JART, Japanese Adult Reading Test; mGAF, modified Global Assessment; PANSS, Positive and Negative Syndrome Scale; SCoRS, Schizophrenia Cognition Rating Scale.

^a^Demographic difference between groups were examined by Analysis of Variance or chi-square test.

^b^Data are ranging from 0 to 10, with larger number representing more worse function.

^c^Data are ranging from 0 to 100. Healthy subjects generally have a score ranging from 90 to 100.

^d^BACS composite score was calculated by averaging all z-scores of the six primary measures from the BACS.Asterisks indicate statistical significance (*p < 0.05, **p < 0.01).

### 3.2 Comparison of ERP data across outcome groups

The ERP results for the four outcome groups are summarized in **Table 2**. P300 and fMMN amplitudes and latencies did not differ significantly between the groups. A significant group effect was observed for dMMN amplitude [F (3,77) = 5.580, p = 0.002]. *Post-hoc* comparisons indicated that the Outcome 2 group exhibited significantly larger (i.e., more negative) dMMN amplitudes than the Outcome 3 and 4 groups. No significant group differences were found in the dMMN latency.

**Table 2:** ERP data.

ERP measures	All	Outcome 1	Outcome 2	Outcome 3	Outcome 4	Statistics
P300						
amplitude	14.1 (5.5)	14.8 (6.4)	14.4 (6.2)	14.8 (5.1)	12.3 (3.9)	F (3,83)=1.029, p=0.384
latency	312.0 (38.2)	311.2 (29.6)	300.8 (45.0)	312.7 (44.4)	321.0 (34.4)	F (3,83)=0.954, p=0.419
dMMN						
amplitude	-5.4 (2.0)	-5.4 (1.4)	-6.9 (2.3)	-5.0 (1.4)	-4.5 (2.1)	F (3,77)=5.580, p=0.002*, Outcome 2<3,4
latency	177.0 (18.5)	173.0 (13.2)	180.9 (22.2)	173.2 (18.0)	182.8 (20.8)	F (3,77)=1.612, p=0.194
fMMN						
amplitude	-4.4 (2.1)	-4.8 (2.7)	-4.7 (1.7)	-4.2 (1.7)	-3.9 (1.9)	F (3,82)=0.943, p=0.424
latency	113.0 (26.3)	118.3 (27.7)	112.3 (28.0)	111.4 (22.6)	109.6 (27.5)	F (3,82)=0.469, p=0.704

ERP data represent peak amplitudes [µV] and latencies [msec] for each group [mean (SD)].

dMMN, duration mismatch negativity; fMMN, frequency mismatch negativity.Asterisks indicate statistical significance (*p < 0.05).

**Figure 1:**
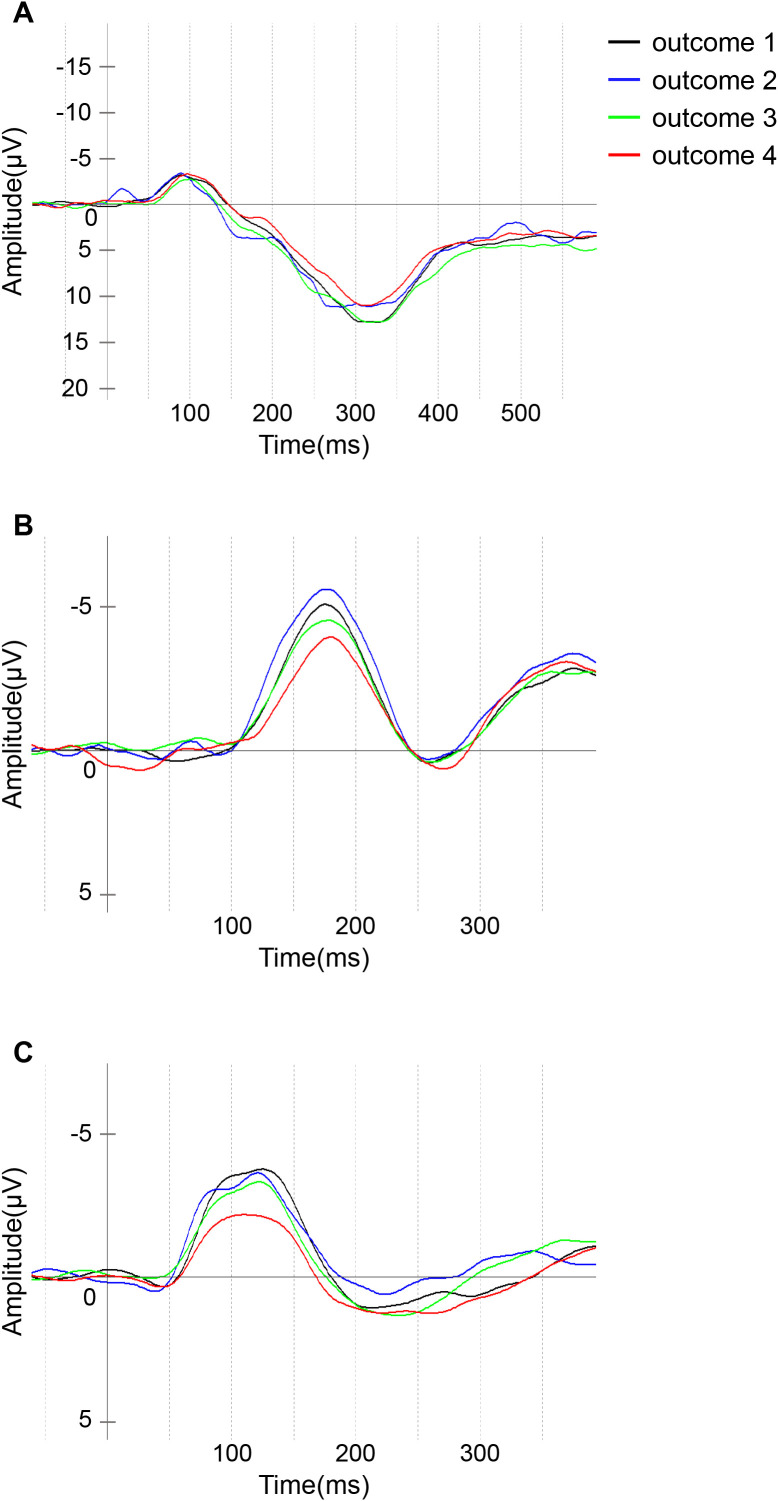
Grand-average ERP waveforms across clinical outcome groups. **(A)** Grand-average P300 waveforms recorded at the Pz electrode for each outcome group. **(B)** Grand-average duration mismatch negativity (dMMN) difference waveforms recorded at the Fz electrode. **(C)** Grand-average frequency mismatch negativity (fMMN) difference waveforms recorded at the Fz electrode. For all panels, waveforms are plotted with negative values upward. dMMN, duration mismatch negativity; ERP, event-related potential; fMMN, frequency mismatch negativity.

The grand-averaged ERP waveforms for P300 at Pz and for dMMN and fMMN at Fz are shown in **Figure 1**.

### 3.3 Ordinal logistic regression results

Ordinal logistic regression was conducted to identify the baseline predictors of long-term clinical outcomes among individuals with ARMS. Variable selection was performed independently across 20 imputed datasets using a bidirectional AIC-based, stepwise procedure. Five predictors met the majority-rule criterion (≥10 of 20 imputations) and were retained in the final pooled model: Unusual Thought Content, dMMN amplitude, SCoRS, fMMN latency, and dMMN latency (**Tables 3A, B**).

Table 3AStepwise variable selection.

**Table:** 

Stepwise variable selection		
Variable	Selected, n (%)	Final_inclusion
Unusual Thought Content	20 (100)	Included
dMMN amplitude	20 (100)	Included
SCoRS	14 (70)	Included
fMMN latency	13 (65)	Not included
dMMN latency	11 (55)	Not included

Number and percentage of imputations in which each variable was selected in the 20 datasets.

**Table 3B:** Ordinal logistic regression model results.

Ordinal logistic regression model results							
Predictor	β	SE	t	p	OR	95% CI for OR [lower – upper]	FMI
Unusual Thought Content	0.495	0.145	3.413	0.001	1.641	1.235-2.180	0.056
dMMN amplitude	-0.236	0.105	-2.244	0.025	0.789	0.642-0.971	0.057
SCoRS	0.216	0.1	2.149	0.032	1.241	1.019-1.510	0.093
fMMN latency	-0.014	0.008	-1.652	0.099	0.986	0.970-1.003	0.08
dMMN latency	0.016	0.011	1.456	0.145	1.016	0.994-1.039	0.074

Model fit indices: McFadden’s Pseudo R²: 0.121 AIC = 226.9; BIC = 246.7 Variables were selected using AIC-based stepwise selection across 20 multiply imputed datasets. Variables selected in ≥50% of imputations were included in the final ordinal logistic regression model, with estimates pooled using Rubin’s rules. AIC, Akaike’s Information Criterion; BIC, Bayesian Information Criterion; β, Regression Coefficients; dMMN, duration mismatch negativity; fMMN, frequency mismatch negativity; FMI, fraction of missing information; OR, odds ratio; SCoRS, Schizophrenia Cognition Rating Scale; SE, standard error.

**Figure 2:**
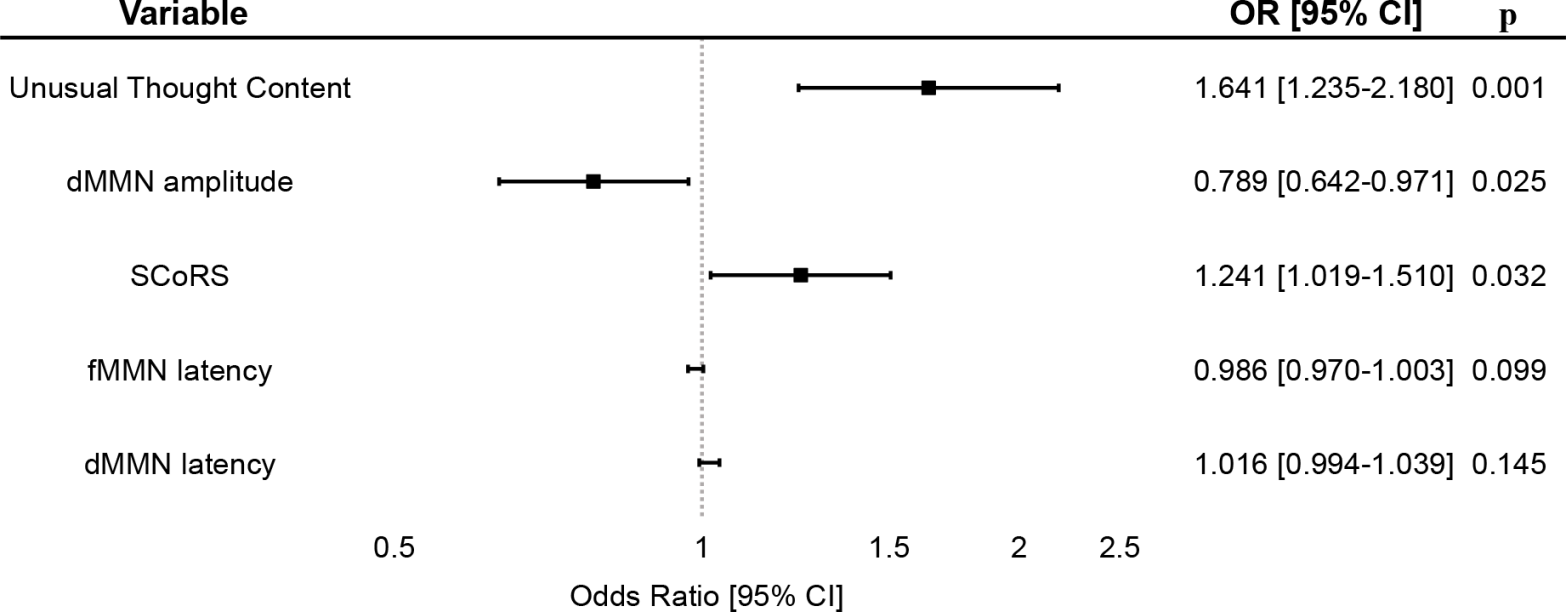
Forest plot of odds ratios for predictors included in the final ordinal logistic regression model. Odds ratios and 95% confidence intervals were estimated using multiple imputation and pooled according to Rubin’s rules. dMMN, duration mismatch negativity; fMMN, frequency mismatch negativity; OR, odds ratio; SCoRS, Schizophrenia Cognition Rating Scale.

In the pooled model based on Rubin’s rules, three variables were identified as significant independent predictors of worse clinical outcomes: higher baseline Unusual Thought Content severity (OR = 1.64, 95% CI: 1.24–2.18, p = 0.001), smaller (i.e., less negative) dMMN amplitude (OR = 0.79, 95% CI: 0.64–0.97, p = 0.025), and higher SCoRS scores (OR = 1.24, 95% CI: 1.02–1.51, p = 0.032). Although fMMN and dMMN latencies met the majority-rule criterion and were included in the final regression model, their coefficients did not reach statistical significance in the pooled analysis (p = 0.099 and p = 0.145, respectively). A forest plot of the ordinal logistic regression results is shown in **Figure 2**.

The model showed a McFadden pseudo-R² of 0.121, with an AIC of 226.9 and a BIC of 246.7. Assessment of the proportional-odds assumption showed that regression coefficients were generally consistent across cumulative thresholds for most predictors included in the final model, supporting the appropriateness of the proportional-odds model. The ordinal C-index across the multiply imputed datasets averaged 0.73. The mean multi-class Brier score was 0.65. Calibration plots showed no major miscalibration between predicted and observed outcome probabilities. Bootstrap-based internal validation showed an average optimism of 0.089 in the C-index. The optimism-corrected C-index was estimated to be 0.641.

Considering the potential influence of antipsychotic medication at baseline, two additional analyses were conducted. First, the ordinal logistic regression analysis restricted to drug-naïve individuals demonstrated that higher baseline Unusual Thought Content severity (OR = 1.79, 95% CI: 1.29–2.50, p = 0.00058) and higher SCoRS scores (OR = 1.33, 95% CI: 1.03–1.70, p = 0.026) remained significantly associated with worse clinical outcomes, while smaller dMMN amplitude showed a trend toward significance (OR = 0.80, 95% CI: 0.64–1.01, p = 0.063). Second, even when baseline antipsychotic use was entered as a forced covariate, the results remained essentially the same; Unusual Thought Content severity (OR = 1.64, 95% CI: 1.24–2.18, p = 0.00061), SCoRS scores (OR = 1.24, 95% CI: 1.02–1.52, p = 0.030), and dMMN amplitude (OR = 0.79, 95% CI: 0.64–0.98, p = 0.028) were significantly associated with outcomes. Overall, these additional analyses indicate that baseline antipsychotic exposure did not materially influence our main findings.

## 4 Discussion

This study examined the predictors of clinical outcomes in individuals with ARMS using an outcome classification framework based on the NAPLS-2, in which clinical outcomes were stratified into four ordered categories. Candidate predictors were derived from baseline assessments including clinical, functional, and electrophysiological measures. Focusing on the heterogeneity of outcomes among individuals who did not transition to psychosis, this approach goes beyond the traditional dichotomous endpoint of transition versus non-transition to capture multiple stages, ranging from remission to persistent symptoms, prodromal progression, and transition to psychosis. Using this multimodal and ordinal framework, baseline dMMN amplitude, Unusual Thought Content severity, and global cognitive functioning assessed by SCoRS were identified as variables associated with ordered clinical outcomes. To the best of our knowledge, no previous study has examined outcome prediction in ARMS using a comparable multimodal and ordinal approach.

### 4.1 dMMN amplitude

MMN is thought to arise from predictive coding mechanisms supported by hierarchical temporal–frontal networks (35, 49), and converging evidence suggests a close association between MMN generation and N-methyl-D-aspartate (NMDA) receptor-mediated synaptic plasticity (50). These mechanisms are consistent with the core pathophysiological features of schizophrenia, such as frontotemporal cortical volume reduction and the NMDA hypofunction hypothesis. Indeed, a reduced MMN amplitude has been consistently reported in schizophrenia and is regarded as a robust neurophysiological finding (51). Furthermore, a meta-analysis demonstrated that dMMN elicits a larger effect size than fMMN, even in first-episode schizophrenia, suggesting that dMMN may be particularly sensitive to early pathophysiological changes (52). These observations underscore the importance of dMMN in clinical and translational studies.

MMN has been widely examined as a potential biomarker for clinical outcomes in ARMS/CHR-P populations. Prospective studies in ARMS/CHR-P populations have consistently demonstrated reduced baseline dMMN amplitudes in individuals who subsequently transitioned to psychosis compared with those who did not (53, 54). Moreover, the observation in the present study that individuals who developed psychosis (Outcome 4) exhibited lower dMMN amplitudes than non-transitioned individuals (Outcomes 1-3) (p = 0.027, data not shown) is in line with prior findings, which provides additional support for the validity of the present results. In addition to transition prediction, several studies have suggested that baseline dMMN amplitude may be associated with symptom improvement and functional recovery among individuals with CHR-P who do not develop psychosis (13, 14). Furthermore, in individuals with ARMS/CHR-P, dMMN amplitude has been reported to correlate with cognitive abilities, particularly verbal fluency (30), as well as with global functioning (55). In patients with schizophrenia, the dMMN amplitude has been shown to predict future cognitive and functional outcomes, as assessed by the Trail Making Test Part B (TMT-B) (56), and to predict remission (11, 33). Together, these findings indicate that dMMN is relevant not only to diagnostic outcomes but also to broader functional outcomes in schizophrenia and ARMS/CHR-P. These results are consistent with those of our ordinal logistic regression analysis, indicating that reduced dMMN amplitude was a significant predictor of poorer future outcomes. Overall, these findings suggest that individuals with ARMS who show unfavorable clinical trajectories have underlying biological vulnerabilities that manifest as an attenuated MMN response.

### 4.2 Unusual thought content

In addition to dMMN, the present study also identified symptom-related predictors of clinical outcomes in ARMS using ordinal logistic analysis. Notably, Unusual Thought Content exhibited the largest effect size among all explanatory variables selected in the regression analysis. Attenuated positive symptoms are considered a central clinical feature of high risk for psychosis and play an important role in diagnostic assessment and outcome prediction (57). Among these symptoms, Unusual Thought Content refers to attenuated delusion-like ideas, including unusual beliefs or interpretations that do not reach full psychotic intensity, such as thought insertion (58).

Previous ARMS/CHR-P research has consistently identified the severity of attenuated positive symptoms as a key predictor of subsequent transition to psychosis. In particular, several clinical prediction models have demonstrated that the baseline severity of Unusual Thought Content significantly contributes to predicting psychosis onset in individuals meeting the ARMS/CHR-P criteria (59–61). Unusual Thought Content is considered to reflect disturbances in self-experience or ego function and has long been recognized as a core psychopathological feature of schizophrenia, corresponding to primary delusions with high diagnostic significance. In line with this conceptualization, a scale of self-alienation-related attributes (Self-A) based on the Minnesota Multiphasic Personality Inventory (MMPI) demonstrated that higher Self-A scores were strongly associated with subsequent transition to psychosis in individuals with ARMS (62). These findings underscore the clinical relevance of Unusual Thought Content as a prognostic indicator in individuals with ARMS/CHR-P. Consistent with this body of evidence, the present study found that the baseline severity of Unusual Thought Content was associated with ordered clinical outcomes in individuals with ARMS, supporting its role as a meaningful predictor across multiple ordered stages of outcome severity.

### 4.3 SCoRS

Cognitive impairment is a well-established core feature of schizophrenia and is observed across multiple cognitive domains, even in patients with relatively preserved intellectual function (63). Similar neurocognitive impairments have also been reported in individuals with ARMS/CHR-P (64–66), indicating that cognitive dysfunction is frequently observed in such individuals irrespective of subsequent psychosis onset. Accumulating evidence suggests that cognitive function plays an important role in determining clinical and functional outcomes in individuals with ARMS/CHR-P (66). In these individuals, baseline neurocognitive impairment has been associated with an increased risk of later transition to psychosis (65–67). Furthermore, cognitive processing speed has been identified as a significant predictor of social functioning 12 months after baseline, with deficits in this domain linked to poor functional trajectories (68).

The SCoRS is an interview-based assessment designed to evaluate cognitive function in relation to everyday performance (26). SCoRS scores have been shown to correlate moderately to strongly with objective neurocognitive test performance and real-world functional outcomes, supporting their utility as clinically meaningful measures of cognition (69). The Japanese version of the SCoRS (SCoRS-J), used in this study, is a validated instrument (25) and has demonstrated strong correlations with objective cognitive performance assessed by the BACS and real-world functioning measured by the Social and Occupational Functioning Assessment Scale (SOFAS), both in patients with schizophrenia and in individuals with ARMS (70).

Consistent with these previous findings, the present study demonstrated that baseline global cognitive functioning assessed using the SCoRS was associated with ordered clinical outcomes in individuals with ARMS. Given that cognitive impairments in ARMS and schizophrenia are relatively stable over time, poorer baseline cognitive functioning may reflect a trait-like vulnerability that predisposes individuals to unfavorable long-term outcomes. Conversely, preserved cognitive function at baseline may serve as a protective factor, contributing to more favorable clinical trajectories (71, 72). Because the SCoRS captures cognitive functioning in relation to everyday life, it may be particularly well suited to predicting graded clinical outcomes beyond simple transition status. Given its interview-based format, high inter-rater reliability, and low burden on both patients and clinicians, the SCoRS offers practical advantages in clinical settings.

### 4.4 Multimodal approaches to outcome prediction

As described above, clinical outcomes in ARMS are not determined by a single domain but instead are thought to be associated with the combined influence of neurophysiological, symptomatic, and functional factors. Based on this view, several studies have attempted to investigate outcome prediction in ARMS/CHR-P populations using multimodal approaches. For example, Schmidt et al. (2017) conducted a simulation-based study examining hypothetical combinations of electrophysiological measures (specifically P300 as an ERP), blood markers, and structural brain imaging findings (73). A more recent study integrated structural brain imaging, cognitive measures, ERPs, and polyunsaturated fatty acid levels (74). In addition, Koutsouleris et al. (2021) reported a multimodal machine-learning workflow that integrated clinical symptoms (e.g., Unusual Thought Content), neurocognitive function, structural brain imaging findings, polygenic risk scores, and clinician ratings, achieving high accuracy in predicting the transition to psychosis (75).

In this context, the outcome predictors identified in the present study encompassed domains that have been repeatedly implicated in both single-modality and multimodal prediction studies, including symptomatic features (Unusual Thought Content), cognitive function, and neurophysiological indices. This convergence with prior findings supports the validity of our results. Importantly, the present study extends previous multimodal work by applying an ordinal outcome framework that captures multiple stages of clinical trajectories beyond simple transition status, thereby providing a more fine-grained characterization of outcome heterogeneity in ARMS/CHR-P populations. Simultaneously, the optimal combination and relative weighting of predictive modalities remain to be established, and further research is required to determine how different domains can be most effectively integrated to improve outcome prediction in ARMS/CHR-P.

### 4.5 Potential influence of baseline antipsychotic exposure

Baseline antipsychotic exposure represents an important methodological consideration in outcome prediction studies of individuals with ARMS. In the present study, additional analyses restricted to drug-naïve individuals and analyses including baseline antipsychotic medication as a covariate yielded results largely consistent with the primary analysis, suggesting that the main findings are unlikely to be explained solely by baseline antipsychotic exposure. In previous studies of CHR-P populations, baseline antipsychotic exposure has been discussed as a potential marker of greater clinical severity rather than a protective effect (76). Furthermore, previous electrophysiological studies have suggested that MMN amplitude is relatively insensitive to antipsychotic dosage, and a recent magnetoencephalography study reported no significant association between antipsychotic exposure and duration-deviant MMN amplitude (17). Taken together, these lines of evidence suggest that the associations between indices and clinical outcomes observed in the present study are unlikely to be explained solely by medication effects. However, the potential influence of antipsychotic exposure cannot be completely excluded and should be interpreted with caution.

### 4.6 Limitation

The present study has several limitations. First, the sample size was relatively small, and all participants were recruited from a single clinical center, which may limit the statistical power and generalizability of the results. Bootstrap-based internal validation demonstrated an average optimism of 0.089 in the C-index, indicating that some degree of overfitting may remain due to the relatively small sample size in relation to the number of candidate predictors. Second, multiple ERP recording systems and electrode caps were used during the data acquisition period. Although identical stimulus presentation parameters and preprocessing procedures were applied across recordings to minimize technical variability, the potential influence of recording system differences cannot be completely excluded. Third, data for several baseline variables were missing. Although multiple imputation was used to retain the available cases, the potential influence of missing data on the results cannot be entirely excluded. Fourth, clinical outcomes were determined based on the final available follow-up assessment, and the follow-up duration varied across participants. Consequently, we cannot exclude the possibility that some individuals may exhibit different clinical outcomes with longer or more uniform follow-up durations. Variations in follow-up periods among participants may have resulted in differences in exposure time to transition risk, which represents a methodological limitation of this study. Future studies using time-to-event modeling approaches may help further refine trajectory-specific prediction in ARMS populations. Fifth, the outcome classification in this study was based on a framework originally developed using the SOPS, whereas the clinical assessments in the present cohort were conducted using the CAARMS. Although the CAARMS and SOPS are closely aligned conceptually and share comparable definitions (29), differences in operational criteria may have affected the outcome categorization. However, previous work has demonstrated a substantial correspondence between the CAARMS- and SOPS-based classifications, and supplementary analyses using converted scores (77) have yielded consistent results (data not shown). Finally, the potential influence of antipsychotic medication cannot be completely excluded. In the present cohort, only a small proportion of participants received antipsychotic medication at baseline, and the antipsychotic dose did not differ significantly across the outcome groups. Moreover, previous studies have shown that MMN amplitude remains relatively stable following antipsychotic treatment (17, 78). Although sub-analyses restricted to drug-naïve individuals and analyses including antipsychotic use as a covariate yielded results broadly consistent with the primary analysis, residual medication effects may still have influenced the observed associations. These limitations highlight the need for replication in larger, multi-center longitudinal cohorts.

### 4.7 Conclusion

In conclusion, the present findings suggest that future clinical outcomes in individuals with ARMS may be predicted using factors spanning multiple domains, including neurophysiological, clinical, and functional measures. Early stratification of clinical outcomes at the ARMS stage may contribute to the development of more individualized early intervention strategies tailored to subsequent clinical courses. Although incorporating a broader range of modalities may further improve predictive accuracy, it is noteworthy that all the modalities selected in the present study were simple and non-invasive, highlighting the practical significance and feasibility of our approach for clinical application.

## Data Availability

The raw data supporting the conclusions of this article will be made available by the authors, without undue reservation.
