# Supplementary material for: Predicting ordinal clinical outcomes in at-risk mental states: a multimodal approach
Source: Front Psychiatry. 2026 May 28;17:1808209. doi: 10.3389/fpsyt.2026.1808209 (PMC13253625; doi:10.3389/fpsyt.2026.1808209)
Supplement: Supplementary file 1 [file DataSheet1.docx]

Suppl 1: Detailed comparison of symptom severity between the assessment tools used in the CAARMS and the SOPS.

Unusual Thought Content

|  | 0 Absent | 1 Questionably Present | 2 Mild | 3 Moderate | 4 Moderately Severe | 5 Severe but Not Psychotic | 6 Severe and Psychotic |
| --- | --- | --- | --- | --- | --- | --- | --- |
| **SOPS** P1: Unusual Thought Content / Delusional Ideas | - | "Mind tricks" that are puzzling. Sense that something is different. | Overly interested in fantasy life. Unusually valued ideas/beliefs. Some superstitions beyond what might be expected by the average person but within cultural norms. | Unanticipated mental events that are puzzling, unwilled, but not easily ignored. Experiences seem meaningful because they recur and will not go away. Functions mostly as usual. | Sense that ideas/experiences/beliefs may be coming from outside oneself or that they may be real, but doubt remains intact. Distracting, bothersome. May affect functioning. | Experiences familiar, anticipated. Doubt can be induced by contrary evidence and others' opinions. Distressingly real. Affects daily functioning. | Delusional conviction (with no doubt) at least intermittently. Interferes persistently with thinking, feeling, social relations, and/or behavior. |
|  | 0 Absent | 1 Questionable | 2 Mild | 3 Moderate | 4 Marked | 5 Severe but Not Psychotic | 6 Psychotic and very severe |
| **CAARMS** Unusual Thought Content | No unusual thought content. | Mild elaboration of conventional beliefs as held by a proportion of the population | Vague sense that something is different, or not quite right with the world, a sense that things have changed but not able to be clearly articulated. Subject not concerned/ worried about this experience. | A feeling of perplexity. A stronger sense of uncertainty regarding thoughts than 2. | Referential ideas that certain events, objects or people have a particular and unusual significance. Feeling that experience may be coming from outside the self. Belief not held with conviction, subject able to question. Does not result in change in behaviour. | Unusual thoughts that contain completely original and highly improbable material. Subject can doubt (not held with delusional conviction), or which the subject does not believe all the time. May result in some change in behaviour, but minor. | Unusual thoughts containing original and highly improbable material held with delusional conviction (no doubt). May have marked impact on behaviour. |

Non-Bizarre Ideas

|  | 0 Absent | 1 Questionably Present | 2 Mild | 3 Moderate | 4 Moderately Severe | 5 Severe but Not Psychotic | 6 Severe and Psychotic |
| --- | --- | --- | --- | --- | --- | --- | --- |
| **SOPS** P2: Suspiciousness / Persecutory Ideas | - | Wariness. | Concerns about safety. Hypervigilance without clear source of danger. | Concerns that people are untrustworthy and/or may harbor ill will. Sense of unease and need for vigilance (often unfocused). Mistrustful. Recurrent (yet unfounded) sense that people might be thinking or saying negative things about person. | Thoughts of being the object of negative attention. Sense that people may wish harm. Self-generated skepticism present. Preoccupying, distressing. May affect daily functioning. May appear defensive in response to questioning. | Beliefs about danger from hostile intentions of others. Skepticism and perspective can prevail with non-confirming evidence or other’s opinion. Anxious, unsettled. Daily functioning affected. Guarded presentation may diminish information gathered in the interview. | Delusional paranoid conviction (no doubt) at least intermittently.　Frightened, avoidant, watchful. Interferes persistently with thinking, feeling, social relations, and/or behavior. |
|  | 0 Absent | 1 Questionable | 2 Mild | 3 Moderate | 4 Marked | 5 Severe but Not Psychotic | 6 Psychotic and very severe |
| **CAARMS** Non-Bizarre Ideas | No unusual thought content. | Subtle changes that could be reality based. Eg. Very self-conscious. | Increased self-consciousness. Eg. Feeling that others look at the subject, or talk about the subject. Or feeling of increased self- importance. Subject able to question. | Odd or unusual thoughts but whose content is not entirely implausible- may be some logical evidence. More evidence than rating of 4. Content of thoughts not original i.e. jealousy, mild paranoia. | Clearly idiosyncratic beliefs, which although ’possible’ have arisen without logical evidence. Less evidence than rating of 3. Eg. Thoughts that others wish the subject harm, which can be easily dismissed. Thoughts of having special powers, which can be easily dismissed. | Unusual thoughts about which there is some doubt (not held with delusional conviction), or which the subject does not believe all the time. May result in some change in behaviour, but minor. | Unusual thoughts containing original and highly improbable material held with delusional conviction (no doubt). May have marked impact on behaviour. |

Grandiosity

|  | 0 Absent | 1 Questionably Present | 2 Mild | 3 Moderate | 4 Moderately Severe | 5 Severe but Not Psychotic | 6 Severe and Psychotic |
| --- | --- | --- | --- | --- | --- | --- | --- |
| **SOPS** Grandiosity | - | Private thoughts of being better than others. | Mostly private thoughts of being talented, understanding, or gifted. | Notions of being unusually gifted, powerful or special and have exaggerated expectations. May be expansive but can redirect to the everyday on own. | Beliefs of talent, influence, and abilities. Unrealistic goals that may affect plans and functioning, but responsive to other’s concerns and limits. | Compelling beliefs of superior intellect, attractiveness, power, or fame. Skepticism and modesty can only be elicited by the efforts of others. Affects functioning. | Delusions of grandiosity with conviction (no doubt) at least intermittently Interferes persistently with thinking, feeling, social relations, or behavior. |
|  | 1 Absent | 2 Minimal | 3 Mild | 4 Moderate | 5 Moderate-severe | 6 Severe | 7 Extreme |
| **PANSS** Grandiosity^1^ | Definition does not apply. | Questionable pathology; may be at the upper extreme of normal limits. | Some expansiveness or boastfulness is evident, but without clear-cut grandiose delusions. | Feels distinctly and unrealistically superior to others. Some poorly formed delusions about special status or abilities may be present but are not acted upon. | Clear-cut delusions concerning remarkable abilities, status or power are expressed and influence attitude but not behaviour. | Clear-cut delusions of remarkable superiority involving more than one parameter (wealth,  knowledge, fame, etc) are expressed, notably influence interactions and may be acted upon. | Thinking, interactions and behaviour are dominated by multiple delusions of amazing  ability, wealth, knowledge, fame, power and/or moral stature, which may take on a bizarre quality. |
|  |  |  |  |  |  |  |  |

Perceptual Abnormalities

|  | 0 Absent | 1 Questionably Present | 2 Mild | 3 Moderate | 4 Moderately Severe | 5 Severe but Not Psychotic | 6 Severe and Psychotic |
| --- | --- | --- | --- | --- | --- | --- | --- |
| **SOPS** Perceptual Abnormalities / Hallucinations | - | Minor, but noticeable perceptual sensitivity (e.g. heightened, dulled, distorted, etc.). | Unformed perceptual experiences/ changes that are noticed but not considered to be significant. | Recurrent, unformed, images (e.g., shadows, trails, sounds, etc.), illusions, or persistent perceptual distortions that are puzzling and experienced as unusual. | Illusions or momentary formed hallucinations that are ultimately recognized as unreal yet can be distracting, curious, unsettling. May affect functioning. | Hallucinations experienced as external to self though skepticism can be induced by others. mesmerizing, distressing. Affects daily functioning. | Hallucinations perceived as real and distinct from the person's thoughts. Skepticism cannot be induced. Captures attention, frightening. Interferes persistently with thinking, feeling, social relations and/or behavior. |
|  | 0 Absent | 1 Questionable | 2 Mild | 3 Moderate | 4 Marked | 5 Severe but Not Psychotic | 6 Psychotic and very severe |
| **CAARMS** Perceptual Abnormalities | No abnormal perceptual experience. | - | Heightened, or dulled perceptions, distortions, illusions (eg lights/ shadows). Not particularly distressing. Hypnogogic/ hypnopompic experiences | More puzzling experiences: more intense/vivid distortions/ illusions, indistinct murmuring, etc. Subject unsure of nature of experiences. Able to dismiss. Not distressing. Derealisation/ depersonalisⁿ | Much clearer experiences than 3 such as name being called, hearing phone ringing etc, but may be fleeting/ transient. Able to give plausible explanation for experience. May be associated with mild distress. | True hallucinations i.e. hearing voices or conversation, feeling something touching body. Subject able to question experience with effort. May be frightening or associated with some distress. | True hallucinations which the subject believes are true at the time of, and after, experiencing them. May be very distressing |

Disorganised Speech

|  | 0 Absent | 1 Questionably Present | 2 Mild | 3 Moderate | 4 Moderately Severe | 5 Severe but Not Psychotic | 6 Severe and Psychotic |
| --- | --- | --- | --- | --- | --- | --- | --- |
| **SOPS** P5: Disorganized Communication | - | Occasional word or phrase doesn’t make sense. | Speech that is slightly vague, muddled, overelaborate or stereotyped. | Incorrect words, irrelevant topics. Goes off track, but redirects on own. | Speech is circumstantial (i.e. eventually getting to the point). Difficulty directing sentences toward a goal. Sudden pauses. Can be redirected with occasional questions and structuring. | Speech tangential (i.e. never getting to the point). Some loosening of associations or blocking. Can reorient briefly with frequent prompts or questions. | Communication persistently loose, irrelevant, or blocked and unintelligible when under minimal pressure or when the content of the communication is complex. Not responsive to structuring of the interview. |
|  | 0 Absent | 1 Questionable | 2 Mild | 3 Moderate | 4 Marked | 5 Severe but Not Psychotic | 6 Psychotic and very severe |
| **CAARMS** Disorganised Speech | Normal logical speech, no disorganisation, no problems communicating or being understood. | - | Slight subjective difficulties eg problems getting message across. Not noticeable by others. | Somewhat vague, some evidence of circumstantiality, or irrelevance in speech. Feeling of not being understood. | Clear evidence of mild disconnected speech and thought patterns. Links between ideas rather tangential. Increased feeling of frustration in conversation. | Marked circumstantiality, or tangentiality in speech, but responds to structuring in interview. May have to resort to gesture, or mime to communicate. | Lack of coherence, unintelligible speech, significant difficulty following line of thought. Loose associations in speech. |

1. The item grandiosity is not assessed in the CAARMS; therefore, the corresponding PANSS positive symptom item for grandiosity was used as a proxy measure.

Abbreviations: CAARMS, Comprehensive Assessment of At-Risk Mental States; PANSS, Positive and Negative Syndrome Scale; SOPS, The Scale of Prodromal Symptoms.

Suppl 2. EEG and MMN settings.

Sampling rate: 500 Hz

Reference: Aav

Bandwidth: 0.53–120 Hz

Notch filter: 60 Hz

Sound stimulus delivery method: through binaural ears, through headphones

Distraction: silent animation

Sound stimulus (through headphones)

|  | P300 block | dMMN block | fMMN block |
| --- | --- | --- | --- |
| No.trials (Standard/Deviant) | 200 / 50 | 1350 / 150 | 1350 / 150 |
| Standard tone | 1000 Hz, 50 ms | 1000 Hz, 50 ms | 1000 Hz, 50 ms |
| Deviant tone | 2000 Hz, 50 ms | 1000 Hz, 100 ms | 1500 Hz, 50 ms |
| Probability (Deviant) | 20% | 10% | 10% |
| ISI / SOA | 1500 ms - 2500 ms (random) | 500 ms / 550 ms (standard) or 600 ms (deviant) | 500 ms / 550 ms |
| Rise/fall time | 10 ms | 10 ms | 10 ms |
| Sound level | 60 dB SPL (binaural) | 60 dB SPL (binaural) | 60 dB SPL (binaural) |
|  |  |  |  |
| Abbreviations: EEG, electroencephalography; dMMN, duration mismatch negativity; fMMN, frequency mismatch negativity; ISI, inter-stimulus interval; SOA, stimulus-onset asynchrony; SPL, sound pressure level. | | | |

Suppl 3. PANSS subscale scores.

|  |  | **All n=87** | **Outcome1 n=25** | **Outcome2 n=18** | **Outcome3 n=21** | **Outcome4 n=23** | **Statistics^a^** |
| --- | --- | --- | --- | --- | --- | --- | --- |
| **PANSS Positive** | Delusions | 2.7(1.3) | 2.4(1.1) | 3.3(1.4) | 2.5(1.4) | 2.8(1.1) | F(3,82)=1.939, p=0.130 |
|  | Conceptual disorganization | 1.4(0.9) | 1.2(0.6) | 1.9(1.6) | 1.2(0.5) | 1.5(0.8) | F(3,82)=2.978, p=0.036*, Outcome1<2 |
|  | Hallucinations | 2.0(1.1) | 1.9(1.1) | 1.9(1.0) | 2.0(1.2) | 2.4(1.3) | F(3,82)=0.861, p=0.465 |
|  | Excitement | 1.1(0.4) | 1.1(0.4) | 1.2(0.7) | 1.1(0.2) | 1.0(0.0) | F(3,82)=1.036, p=0.381 |
|  | Grandiosity | 1.1(0.5) | 1.0(0.0) | 1.0(0.0) | 1.2(0.8) | 1.2(0.6) | F(3,82)=1.617, p=0.192 |
|  | Suspiciousness/persecution | 2.2(1.2) | 1.8(1.1) | 2.9(1.3) | 2.1(1.2) | 2.1(1.1) | F(3,82)=3.388, p=0.022*****, Outcome1<2 |
|  | Hostility | 1.2(0.6) | 1.1(0.4) | 1.5(1.1) | 1.1(0.4) | 1.2(0.5) | F(3,82)=1.813, p=0.151 |
| **PANSS Negative** | Blunted affect | 2.8(1.5) | 2.6(1.7) | 3.1(1.5) | 2.5(1.5) | 2.9(1.2) | F(3,82)=0.644, p=0.589 |
|  | Emotional withdrawal | 2.5(1.4) | 2.4(1.7) | 3.0(1.4) | 2.2(1.3) | 2.6(1.3) | F(3,82)=1.045, p=0.377 |
|  | Poor rapport | 2.0(1.4) | 2.2(1.6) | 2.4(1.6) | 1.6(1.1) | 2.1(1.0) | F(3,82)=1.273, p=0.289 |
|  | Passive/apathetic social withdrawal | 3.3(1.5) | 3.4(1.6) | 3.3(1.3) | 3.0(1.7) | 3.4(1.4) | F(3,82)=0.455, p=0.722 |
|  | Difficulty in abstract thinking | 2.2(1.2) | 2.2(1.2) | 2.0(1.3) | 2.0(0.9) | 2.6(1.4) | F(3,82)=1.039, p=0.380 |
|  | Lack of spontaneity and flow of conversation | 2.5(1.3) | 2.4(1.4) | 2.7(1.3) | 2.1(1.1) | 2.8(1.4) | F(3,82)=1.277, p=0.288 |
|  | Stereotyped thinking | 1.2(0.6) | 1.2(0.6) | 1.3(1.0) | 1.1(0.5) | 1.1(0.3) | F(3,82)=0.583, p=0.628 |
| **PANSS General Psychopathology** | Somatic concern | 1.6(0.9) | 1.5(0.9) | 1.7(1.1) | 1.4(0.9) | 1.7(0.9) | F(3,82)=0.614, p=0.608 |
|  | Anxiety | 2.9(1.2) | 2.4(1.1) | 3.1(1.2) | 2.9(1.2) | 3.2(1.1) | F(3,82)=2.457, p=0.069 |
|  | Guilt feelings | 2.0(1.4) | 1.6(0.9) | 2.5(1.7) | 2.1(1.5) | 2.1(1.4) | F(3,82)=1.668, p=0.180 |
|  | Tension | 1.7(1.0) | 1.7(0.9) | 1.7(1.2) | 1.6(0.9) | 2.0(1.1) | F(3,82)=0.707, p=0.550 |
|  | Mannerisms and Posturing | 1.4(0.8) | 1.1(0.4) | 1.5(0.9) | 1.2(0.6) | 1.6(1.0) | F(3,82)=2.444, p=0.070 |
|  | Depression | 3.2(1.5) | 2.8(1.6) | 3.4(1.2) | 3.1(1.8) | 3.4(1.4) | F(3,82)=0.733, p=0.535 |
|  | Motor retardation | 2.5(1.3) | 2.5(1.5) | 3.0(1.1) | 2.1(1.2) | 2.4(1.2) | F(3,82)=1.779, p=0.158 |
|  | Uncooperativeness | 1.1(0.3) | 1.0(0.2) | 1.2(0.5) | 1.1(0.2) | 1.1(0.3) | F(3,82)=0.663, p=0.577 |
|  | Unusual thought content | 1.8(1.2) | 1.6(1.1) | 2.0(1.1) | 1.8(1.2) | 1.9(1.4) | F(3,82)=0.417, p=0.741 |
|  | Disorientation | 1.1(0.3) | 1.0(0.2) | 1.1(0.5) | 1.1(0.4) | 1.1(0.2) | F(3,82)=0.229, p=0.876 |
|  | Poor attention | 2.3(1.1) | 2.2(1.1) | 2.8(1.2) | 1.8(1.0) | 2.4(1.0) | F(3,82)=2.596, p=0.058 |
|  | Lack of judgment and insight | 1.9(1.2) | 1.8(1.3) | 1.8(1.3) | 1.9(1.0) | 1.9(1.2) | F(3,82)=0.003, p=1.000 |
|  | Disturbance of volition | 1.9(1.1) | 1.7(0.9) | 1.9(1.1) | 2.0(1.4) | 2.0(1.2) | F(3,82)=0.235, p=0.872 |
|  | Poor impulse control | 1.2(0.6) | 1.2(0.6) | 1.2(0.7) | 1.3(0.6) | 1.2(0.7) | F(3,82)=0.152, p=0.928 |
|  | Preoccupation | 1.4(0.9) | 1.3(0.7) | 1.6(1.2) | 1.2(0.6) | 1.6(1.2) | F(3,82)=0.718, p=0.544 |
|  | Active social avoidance | 2.6(1.7) | 2.3(1.6) | 3.5(1.7) | 2.4(1.7) | 2.5(1.4) | F(3,82)=2.257, p=0.088 |

Values are shown as means (standard deviations).

Abbreviations: PANSS, Positive and Negative Syndrome Scale.
